# Effects of high aerobic intensity training in patients with schizophrenia—A controlled trial

**DOI:** 10.3109/08039488.2011.560278

**Published:** 2011-02-18

**Authors:** Jørn Heggelund, Geir E Nilsberg, Jan Hoff, Gunnar Morken, Jan Helgerud

**Affiliations:** 1 Department of Neuroscience, Faculty of Medicine, Norwegian University of Science and Technology, Trond-heim, Norway; Department of Research and Development (AFFU), Division of Psychiatry, St. Olavs University Hospital, Trondheim, Norway; Department of Østmarka, Division of Psychiatry, St. Olavs University Hospital, Trondheim, Norway; 2 Department of Østmarka, Division of Psychiatry, St. Olavs University Hospital, Trondheim, Norway; 3 Department of Circulation and Medical Imaging, Faculty of Medicine, Norwegian University of Science and Technology, Trondheim, Norway; Department of Physical Medicine and Rehabilitation, St.Olavs University Hospital, Trondheim, Norway; 4 Department of Neuroscience, Faculty of Medicine, Norwegian University of Science and Technology, Trondheim, Norway; Department of Østmarka, Division of Psychiatry, St. Olavs University Hospital, Trondheim, Norway; 5 Department of Circulation and Medical Imaging, Faculty of Medicine, Norwegian University of Science and Technology, Trondheim, Norway; Hokksund Medical Rehabilitation Centre, Norway; Telemark University College, Department of Sports and Outdoor Life Studies, Bø, Norway

**Keywords:** Cardiovascular disease, Exercise, Oxygen consumption, Schizophrenia

## Abstract

*Background:* Patients with schizophrenia have a high risk of cardiovascular disease (CVD). High aerobic intensity training (HIT) improve peak oxygen uptake (VO_2peak_), net mechanical efficiency of walking and risk factors for CVD but has not been investigated in patients with schizophrenia. *Aims:* To investigate effects from HIT on VO_2peak_, net mechanical efficiency of walking and risk factors for CVD in patients with schizophrenia. *Methods:* 25 inpatients (F20-29, ICD-10) were allocated to either HIT or playing computer games (CG), 3 days per week for 8 weeks. HIT consisted of 4 × 4-min intervals with 3-min break periods, at 85-95% and 70% of peak heart rate, respectively. *Results:* 12 and seven patients completed HIT and CG, respectively. The baseline VO_2peak_ in both groups combined (*n* = 19) was 36.8 ± 8.2 ml/kg/min and 3.12 ± 0.55 1/min. The HIT group improved VO_2peak_ by 12% from 3.17 ± 0.59 to 3.56 ± 0.68 1/min (*P* < 0.001), more than the CG group (*P* = 0.014). Net mechanical efficiency of walking improved by 12% in the HIT group from 19.8 ± 3.0% to 22.2 ± 4.5% (*P* = 0.005), more than the CG group (*P* = 0.031). The psychiatric symptoms, expressed as the Positive and Negative Syndrome Scale (PANSS) and the Calgary Depression Scale for Schizophrenia (CDSS), did not improve in either group. *Conclusions:* VO_2peak_ and net mechanical efficiency of walking improved significantly by 8 weeks of HIT. HIT should be included in rehabilitation in order to improve physical capacity and contribute risk reduction of CVD.

Patients suffering from schizophrenia have an illnessrelated vulnerability to adopt deleterious lifestyles in addition to a congestion of cardiovascular and metabolic risk factors from antipsychotics and a genetic vulnerability for developing cardiovascular disease (CVD) (1). The combination of these factors causes impaired physical fitness, weight gain, obesity, hyperglycemia, type 2 diabetes, hyperlipidemia and ultimately increased risk of CVD (2, 3). Although these risk factors are related to the physical fitness level and are improvable with aerobic endurance training, there has not been much attention to the contribution of poor physical fitness to the risk of CVD in schizophrenia. Reduced physical fitness also compromises the ability to perform lifestyle physical activity that is necessary for social rehabilitation and integration into the community. Effective aerobic endurance training has the potential to reduce the high risk of CVD, and improve functional ability and quality of life for patients with schizophrenia, as have been reported in other populations with reduced physical fitness (4). Positive psychological effects on mental health and well-being are also reported in patients with schizophrenia (5).

Physical fitness, measured as peak oxygen uptake (VO_2peak_), appears to have more influence on risk of CVD compared with the physical activity level (6). Relatively small improvement in VO_2peak_ is associated with a fair-sized decrement in risk of all-cause mortality and CVD (7). This effect might be induced by an improvement in major risk factors for CVD (8), but physically active people have reduced cardiovascular mortality even when CVD risk factors are present (9).

High aerobic intensity training (HIT) performed as 4 × 4-min intervals are found to be an effective training method to improve VO_2peak_ for healthy individuals, patients with CVD and metabolic syndrome (10–13). Studies on patients with schizophrenia have usually investigated low-intensity exercise corresponding to <70% peak heart rate (HR_peak_) (14-17). Only a few studies have applied direct measurements of oxygen uptake (18-20), but the exact level of VO_2peak_ are not described.

A major concern over the cardiovascular health in people with schizophrenia is their ability to engage in and participate in physical training. In addition, inherent difficulties in recruitment to randomized controlled trials and distrust in methodological issues such as randomization has been described (15, 21).

## Aims

The objectives of the study were to investigate effects from HIT, compared with physical inactivity in the form of playing computer games (CG), in patients suffering from schizophrenia. The primary outcome was changes in VO_2peak_ and net mechanical efficiency of walking. The secondary outcome was effects on other CVD risk factors and symptoms of schizophrenia. We hypothesized that >80% adherence to 24 training sessions with HIT during 8 weeks would improve the primary outcome variables more than the same amount of time spend on CG.

## Materials and Methods

All inpatients at three wards in a University hospital that fulfilled the inclusion criteria were evaluated for eligibility for the study by the medical doctor. In total 38 patients were evaluated, one did not meet the inclusion criteria, five did not want to participate and seven did not participate for other reasons. The first 16 consecutive patients were included in the HIT group and the next nine consecutive patients were included in the CG group. The inclusion criteria were ICD-10 schizophrenia, schizotypal and delusional disorders (F20-F29). Patients were on stable antipsychotic medication for 6 weeks prior to inclusion. Exclusion criteria were coronary artery disease, chronic obstructive pulmonary disease, unstable pharmacological treatment during the intervention period, and not being able to perform physical treadmill testing and exercise.

### Interventions

The HIT group trained 4 × 4-min interval training on a treadmill (Tung Keng Enterprise CO., Ltd, Taiwan) at 85-95% HR_peak_ interspersed with 3 min of active resting periods at a work load corresponding to 70% HR_peak_ between each interval, previously shown to be highly effective (10-13). Patients performed the intervals walking or running with a minimum of 5% inclination. Heart rate was assessed continuously during exercise, using a Polar S610i heart rate monitor (Polar Electro, Finland). The exercise physiologist ensured that the patients performed intervals at the scheduled intensity.

The CG group spent the same amount of time, 36 min three times per week, training to improve their ability in the computer game, Tetris (THQ Inc. Calabasas Hills, CA) using an Xbox Video Game Systems (Microsoft Corporation, Redmond, USA).

Patients performed the training sessions three times per week for 8 weeks. In both groups, training sessions were lead and monitored by the same exercise physiologist. Adherence to at least 19 training sessions (80%) during the 8-week period was required to be included in the effect analyses.

### Testing

The test started with a 10-min warm-up at approximately 50-60% of VO_2peak_. Patients then walked for 6 min on the treadmill at an inclination and speed corresponding to 60 Watt (22, 23). We obtained the measurements of pulmonary gas exchange and heart rate between 5 and 5½ min walking, using the Cortex Metamax II portable metabolic test system (Cortex Biophysik GmbH, Leipzig, Germany) and the Polar S610i heart rate monitor, respectively. Net mechanical efficiency of walking is defined as the percentage of the work input (kilocalories) that is converted into work output. The equation for this calculation is described elsewhere (22, 23).

After testing net mechanical efficiency of walking the patient immediately proceeded with the VO_2peak_ testing protocol. The speed or the inclination was increased every minute to a level that brought the patient to exhaustion in 3-6 min. VO_2peak_ was accepted when VO_2_ leveled off, despite further increases in speed and when respiratory exchange ratio (RER) was above 1.10 (24). The highest heart rate recorded during the last minute of the test was determined as HR_peak_.

The patients took the pre and post-test fasting blood samples in the morning and we calculated low-density lipoprotein (LDL)-cholesterol using the Friedewald equation (25).

Possible changes in positive and negative symptoms were assessed using the Positive and Negative Syndrome Scale (PANSS) (26). The test was performed by two psychiatric nurses, trained in using PANSS, working in the ward and had a personal knowledge of the patient. The Calgary Depression Scale for Schizophrenia (CDSS) was used to assess depressive symptoms (27). The 36-item short form (SF-36) was used to assess the physical health and mental health aspect of health-related quality of life (HRQOL) (28). The SF-36 is a 36-item self-report instrument.

Patients were given 10-min to learn the Tetris computer game before testing. Thereafter patients had three attempts, and the best of three trials were used in the results. We measured performance as total number of lines achieved during a game,

We accomplished this test for the CG, but not for HIT patients. Testing was not done blinded to allocation.

The study was approved by the National Committee for Medical and Health Research Ethics, Middle Norway. The approval number is: 4.2005.1507. ClinicalTrials.gov Identifier: NCT00286299.

### Statistics

We suggested a change in VO_2peak_ from pre- to post-test of 4 ± 3 ml/kg/min for use in the sample size estimation; 18 patients was needed in the study for 80% power to detect a between group difference at *P* < 0.05. Data are expressed as mean and standard deviation (s). Difference in changes from pre- to post-test between groups is expressed as mean difference and 95% confidence intervals (CI). Paired samples *t*-test was performed to determine changes from pre- to post-intervention. Independent sample *t*-test was performed to test differences at baseline and changes from pre- to post-test between the two groups. The significance level (α) was set at *P* < 0.05 (two-tailed).

## Results

During the training period, six of the 25 included patients did not complete the study and are not included in the results: one HIT and one CG patient were discharged from the hospital before completion and one HIT patient was lost because of ankle pains during running. One CG patient disappeared. In addition, two of the HIT patients were excluded because they completed less than 80% of the training sessions. The patients that discontinued the study were not significantly different in any of the measured baseline variables, compared with the patients that completed the study. Patients in the HIT (*n* = 12) and CG (*n* = 7) groups performed 85 ± 9% and 83 ± 6% of the scheduled training sessions, respectively. Characteristics of the patients are presented in Table 1.

**Table 1:** Characteristics of the patients.

	High aerobic intensity training (*n* = 12)	Computer game training (*n* = 7)	All (*n* = 19)
Men/women, *n*	9/3	4/3	13/6
Age (years), mean ± *s*	30.5 ± 8.7	38.9 ± 11.4	33.6 ± 10.3
Age at first contact with psychiatric services	24.8 ± 9.0	25.2 ± 6.3	24.9 ± 7.9
(years), mean ± *s*			
Months of hospitalization, mean ± *s*	35.2 ± 19.6	70.1 ± 62.9	48.0 ± 43.0
ICD-10 diagnosis, *n*			
Schizophrenia	11	6	17
Delusional disorder	1	0	1
Schizoaffective disorder	0	1	1

*s*, standard deviation.

No significant differences between the groups (*P* < 0.05).

After 8 weeks of training, the HIT group improved VO_2peak_ more than the CG group (Table 2). VO_2peak_ increased in the HIT group from pre- to post-test (12%, *P* < 0.001), but no change was apparent in the CG group. In both groups combined (*n* = 19), VO_2peak_ was 36.8 ± 8.2 ml/kg/min and 3.12 ± 0.55 1/min at baseline.

**Table 2:** Physiological variables measured during peak treadmill exercise.

	High aerobic intensity training (*n* = 12)		Computer game training (*n* = 7)		Difference pre-post between groups
			
	Pre	Post	Pre	Post	Mean (95% CI)
VO_2peak_ (l/min), mean ± *s*	3.17 ± 0.59	3.56 ± 0.68***	3.03 ± 0.51	3.09 ± 0.57	0.33 (0.07 to 0.58)†
VO_2peak_ (ml/kg / min), mean ± *s*	36.0 ± 7.4	40.2 ± 6.6***	38.3 ± 9.8	37.9 ± 9.9	4.7 (1.8 to 7.6)††
HR (beats/min), mean ± *s*	175 ± 14	172 ± 15	166 ± 14	167 ± 14	−4 (−11 to 3)
V_E_ (l/min), mean ± *s*	95.8 ± 21.0	103.0 ± 17.1	91.2 ± 19.9	94.7 ± 22.1	3.7 (−6.5 to 13.9)
RER, mean ± *s*	1.11 ± 0.08	1.12 ± 0.06	1.10 ± 0.10	1.12 ± 0.10	0.00 (−0.06 to 0.05)

*s*, standard deviation; VO_2peak_, peak oxygen uptake; HR, heart rate; V_E_, total pulmonary ventilation; RER, respiratory exchange ratio; CI, confidence interval.

*** *P* < 0.001, changes from pre- to post-test.

† *P* < 0.05

†† *P* < 0.01 differences in changes from pre- to post-test between groups.

The HIT group improved net mechanical efficiency of walking more than the CG group (*P* = 0.031, Table 3). The net mechanical efficiency of walking improved from pre- to post-test in the HIT group (12%, *P* = 0.005), but no change was apparent in the CG group. In both groups combined (*n* = 19), the net mechanical efficiency of walking was 19.6 ± 2.9% at baseline.

**Table 3:** Physiological variables measured during 60-Watt submaximal treadmill walking.

	High aerobic intensity training (*n* = 12)		Computer game training (*n* = 7)		Difference pre-post between groups
			
	Pre	Post	Pre	Post	Mean (95% CI)
*ε_net_* (%), mean ± *s*	19.8 ± 3.0	22.2 ± 4.5**	19.4 ± 3.0	19.4 ± 2.5	2.4 (0.3 to 4.6)†
VO_2_ (1/min), mean ± *s*	1.79 ± 0.32	1.70 ± 0.31**	1.77 ± 0.49	1.77 ± 0.45	−0.09 (−0.17 to −0.01)†
VO_2_ (ml/kg/min), mean ± *s*	20.0 ± 2.6	19.3 ± 2.5**	21.5 ± 3.5	21.1 ± 3.2	−0.3 (−1.2 to 0.5)
HR (beats/min), mean ± *s*	140 ± 16	133 ± 15**	136 ± 17	133 ± 11	−4 (−12 to 4)
V_E_ (1/min), mean ± *s*	43.9 ± 10.0	40.9 ± 8.4	45.5 ± 15.7	44.8 ± 12.8	−2.4 (−7.4 to 2.7)
RER, mean ± *s*	0.89 ± 0.06	0.91 ± 0.05	0.93 ± 0.04	0.92 ± 0.04	0.03 (−0.03 to 0.08)

*s*, standard deviation; VO_2_, oxygen uptake; HR, heart rate; V_E_, total pulmonary ventilation; RER, respiratory exchange ratio; *ε_nef_* net mechanical efficiency of walking; CI, confidence interval.

** *P* < 0.01, changes from pre- to post-test.

† *P* < 0.05 difference in changes from pre- to post-test between groups.

A between group change in high-density lipoprotein (HDL)-cholesterol was observed (0.18 mmol/1, *P* = 0.007; Table 4). HDL-cholesterol decreased by 8 ± 8% in the CG group (0.13 mmol/1, *P* = 0.044).

**Table 4:** Hematological values and blood pressure.

	High aerobic intensity training (*n* = 12)		Computer game training (*n* = 7)		Difference pre-post between groups
			
	Pre	Post	Pre	Post	Mean (95% CI)
Body weight pretest (kg), mean ± *s*	90.1 ± 17.9	89.1 ± 16.6	85.3 ± 29.7	87.1 ± 29.9	− 2.8 (− .2 to 0.5)
BMI (kg/m2), mean ± *s*	28.8 ± 4.7	28.5 ± 4.5	27.6 ± 8.5	28.2 ± 8.8	− 0.9 (− 2.0 to 0.1)
Systolic pressure (mmHg), mean ± *s*	131 ± 22	135 ± 21	124 ± 22	128 ± 28	0 (− 15 to 16)
Diastolic pressure (mmHg), mean ± *s*	82 ± 10	84 ± 12	80 ± 12	79 ± 12	3 (− 5 to 12)
Triglyceride (mmol/l), mean ± *s*	1.5 ± 0.9	1.9 ± 1.3	1.8 ± 1.3	1.8 ± 1.2	0.3 (− 0.3 to 1.0)
HDL-cholesterol (mmol/l), mean ± *s*	1.21 ± 0.38	1.26 ± 0.30	1.37 ± 0.38	1.24 ± 0.28**	0.18 (0.06 to 0.31)††
LDL-cholesterol (mmol/l), mean ± *s*	3.15 ± 0.86	2.82 ± 0.73	3.10 ± 0.67	2.95 ± 0.84	− 0.17 (− 0.67 to 0.33)
Hs-CRP (mg/l), mean ± *s*	8.09 ± 18.15	2.67 ± 2.11	4.41 ± 5.34	4.26 ± 5.09	− 5.28 (− 18.83 to 8.28)
Total cholesterol (mmol/l), mean ± *s*	5.0 ± 0.8	4.9 ± 1.0	5.3 ± 0.4	5.0 ± 0.7	0.1 (− 0.5 to 0.7)
Glucose (mmol/l), mean ± *s*	5.5 ± 0.8	5.6 ± 0.6	6.4 ± 2.8	6.8 ± 3.7	− 0.3 (− 1.1 to 0.5)

*s*, standard deviation; BMI, body mass index; HDL, high-density lipoprotein; LDL, low-density lipoprotein; hs-CRP, high-sensitivity serum C-reactive protein; CI, confidence interval.

** *P* < 0.01, changes from pre- to post-test.

†† *P* < 0.01 difference in changes from pre- to post-test between groups.

No significant changes were observed between or within the two groups in PANSS, CDSS and SF-36 (Table 5). The CG group improved their performance in Tetris, measured as total number of lines achieved, from 10 ± 11 lines at pre-test to 83 ± 41 lines at post-test (*P* = 0.003).

**Table 5:** Psychiatric symptoms and quality of life before and after interventions.

	High aerobic intensity training (*n* = 12)		Computer game training (*n* = 7)		Difference pre-post between groups
			
	Pre	Post	Pre	Post	Mean (95% CI)
Total PANSS	74.7 ± 20.9	73.3 ± 24.3	63.4 ± 20.4	61.4 ± 18.9	2.4 (− 13.2 to 18.1)
Total positive, mean ± *s*	16.5 ± 6.9	15.2 ± 6.3	14.6 ± 6.1	13.7 ± 7.4	1.4 (− 1.7 to 4.5)
Total negative, mean ± *s*	20.3 ± 8.2	23.1 ± 10.0	15.9 ± 10.3	15.3 ± 9.8	3.3 (− 2.7 to 9.3)
Total global psychopathology, mean ± *s*	37.8 ± 10.3	35.0 ± 10.7	33.0 ± 7.2	32.4 ± 7.2	− 2.2 (− 10.7 to 6.4)
Total CDSS, mean ± *s*	2.0 ± 2.3	1.9 ± 3.4	4.2 ± 2.4	3.7 ± 1.6	0.4 (− 1.6 to 2.4)
SF-36					
Physical health, mean ± *s*	52.8 ± 7.7	52.3 ± 7.7	45.7 ± 8.3	47.6 ± 5.6	− 2.4 (− 6.8 to 2.1)
Mental health, mean ± *s*	45.8 ± 10.2	44.9 ± 10.9	44.5 ± 10.5	47.7 ± 2.2	− 4.1 (− 13.9 to 5.8)

*s*, standard deviation; PANSS, Positive and Negative Syndrome Scale; CDSS, Calgary Depression Scale for Schizophrenia; SF-36, 36-item short form; CI, confidence interval. No significant differences in changes from pre- to post-test between or within the two groups.

## Discussion

The primary finding is that the patients suffering from schizophrenia were able to participate in high aerobic intensity training and improve their VO_2peak_. The HIT group improved VO_2peak_ by 12%. The size of the improvement in VO_2peak_ is in line with effects of 8 weeks of training in healthy controls and in patients with CVD (10-12, 29). It seems fair to conclude that, in line with what has been shown for healthy subjects, patients suffering from schizophrenia also benefit from 8 weeks with HIT.

The ability to improve VO_2peak_ is highly related to the ability to adhere to the exercise training, which is considered challenging for patients suffering from schizophrenia. The patients included in the present study were inpatients that suffered from severe schizophrenia, the majority of the patients had several years of hospitalization and some were described as treatment resistant. In spite of the severity of their illness, they managed to participate in HIT and improve VO_2peak_ within a short period. This effectiveness could have important implications for the long-term treatment and prevention of low VO_2peak_.

In both groups combined, the VO_2peak_ at inclusion was low and can be considered close to normative VO_2peak_ for sedentary people of the same age but well below normative values for active healthy people that participate in occasional aerobic exercise ≤2 times per week (30, 31). Low VO_2peak_ is associated with higher risk of cardiovascular morbidity, obesity, high blood pressure, high total- and LDL-cholesterol levels, and reduced glycemic control. A resent meta-analysis defined 28 ml/kg/min (7.9 MET) as a critical level, as those with lower than 28 ml/kg/min had substantial higher rates of all-cause mortality and CVD events compared with those with higher VO_2peak_ (7). Fortunately, only a modest level of improvement in VO_2peak_ appears to confer a significant protective effect from CVD risk factors. Kodama et al. (7) found that every 3.5 ml/kg/min increase in VO_2peak_ was associated with 13% and 15% decrements in risk of all-cause mortality and CVD, respectively. These benefits may result from an improvement in CVD risk factors, but could also be related to other indirect protective mechanisms. The present study found an improvement of 4.2 ml/kg/min after 8 weeks with HIT, and theoretically a considerable reduction in the CVD risk.

VO_2peak_ values are seldom reported in patients suffering from schizophrenia. Carlsson et al. (18, 19) measured oxygen uptake (VO_2_) in people with schizophrenia in the late 1960s. Carlsson et al. (18) presented VO_2_ values that did not seem to reach VO_2peak_ in all patients and are therefore considerable lower than the current VO_2peak_ findings. A recent 3-month intervention study found +5% and -3% change in VO_2peak_ in the exercise group and non-exercise group, respectively (20). The exact level of VO_2peak_ was not reported.

Participation in HIT was associated with improved net mechanical efficiency of walking, compared with the CG. The HIT group improved net mechanical efficiency of walking from 19.8 ± 3.1% to 21.9 ± 4.4%, whereas no significant change was observed within the CG group. This is in line with studies reporting improved work economy both in students and in patients with post-infarction heart failure after participation in walking and running interventions (10, 12). Walking is an important part of daily function and interventions that improve net mechanical efficiency of walking might have great impact in the performance on daily tasks and physical activity in general.

The baseline value for net mechanical efficiency of walking in all patients (*n* = 19) was 19.6%. This is similar to values found in patients with coronary artery disease (19.2%) and lower than in healthy controls (24.7%) (32). It could be assumed that patients with schizophrenia walk with mechanical inefficiency. Psychomotor slowing has been recognized as a feature in people with schizophrenia and probably contributes to walking mechanical inefficiency (33). However, it also seems connected to inactivity, as we found that net mechanical efficiency of walking could be improved after participation in HIT.

The poor VO_2peak_ and net mechanical efficiency of walking described in this study might have adverse effects that could hamper rehabilitation and reintegration into the community, reduce compliance to other treatment regimens, increase stigmatization and discrimination, have harmful effects on quality of life and physical functioning, and increase financial burden (4, 34). Poor VO_2peak_ and net mechanical efficiency of walking involves that lifestyle physical activity, such as walking and household chores will demand a higher percentage of the patients VO_2peak_, making lifestyle physical activity less pleasant. Improved endurance performance is shown to improve functional status in other populations (4). Effective HIT might help the patients to better function in general and cope better with their illness.

The baseline BMI score in all patients was 28.3 kg/m^2^, which is classified as overweight with increased health risk. Blood pressure, triglyceride, hs-CRP, total cholesterol and glucose were within normal values. During the intervention period, HDL-cholesterol changed significantly between the groups.

No significant changes in PANSS, CDSS or in SF-36 in either group or between groups were found in the present study. A few exercise studies have applied the same instruments in patients with schizophrenia. In a study by Beebe et al. (15) the patients participated in less strenuous training that continued for twice as long (16 weeks) as in the present study, or a control group that did not exercise. They reported a non-significant tendency of improvement in total PANSS score in the experimental group. Another study found that total PANSS improved somewhat in a cycling exercise group (−9%) and worsened in a non-exercise group (+13%) after 3 months (20). A significant reduction in depression has been found after an aerobic exercise intervention in a combined group of patients suffering from schizophrenia and bipolar disorder, which could possibly interfere with the result (14). Another study found a significant reduction in overall psychopathology using the Psychiatric Assessment Scale (PAS), Nurses Global Impressions Scale (NGI) and Symptom Checklist-90 (SCL-90) (16). The intervention included a combination of different types of sport activities, meditation and stress education. Thus, it was not possible to separate effects from aerobic exercise alone. Using qualitative measures, studies have reported mood-elevating effects and reduction in depression and anxiety (14, 34). In conclusion, both the type and duration of the intervention along with the type of instruments used to evaluate potential benefits might explain the diverging effects on symptoms of schizophrenia. In patients with chronic schizophrenia already receiving much psychosocial support, it might be difficult to demonstrate improvement in symptoms or in psychosocial function with an 8-week physical training intervention.

A limitation of the study is that we did not conduct a random allocation of subjects to the two groups, but included consecutive patients first to the HIT group and then to the CG group. Some patients with schizophrenia have distrust in randomization, and randomized controlled trials might exclude patients with a high level of symptoms from participating in studies evaluating this type of intervention in schizophrenia (21). To recruit patients, we had to explain very carefully and in detail what they were going to take part in. In this study, a randomized protocol would probably exclude the patients with severe hallucinations or delusions. All patients were able to participate in both interventions and none of them was selected because they fitted one of the groups more than the other. However, as they were specifically asked to participate in a either HIT or CG, their personal preference for that particular intervention might influenced their decision. A selection bias cannot be ruled out and thus compromising the external validity.

This was an efficacy trial, and explored if HIT could work under ideal circumstances. The patients that adhere to less than 80% of the training sessions were excluded. The results can be generalized only to patients who adhere to the HIT. Additionally, the 8-week intervention period is a short period.

## Conclusion

This study indicates that patients with schizophrenia have a level of VO_2peak_ that is associated with increased risk of CVD. The patients also seem to have poor net mechanical efficiency of walking and spend excessive energy during walking. HIT can effectively improve VO_2peak_ and net mechanical efficiency of walking during 8 weeks in patients that adhere to training. Treatment for schizophrenia should include efficient aerobic endurance training to contribute risk reduction of inactivity-related comorbidity and poor physical ability.
